# High CD204^+^ tumor-associated macrophage density predicts a poor prognosis in patients with clear cell renal cell carcinoma

**DOI:** 10.7150/jca.91928

**Published:** 2024-01-21

**Authors:** Yuxia Xie, Guojun Tang, Ping Xie, Xiao Zhao, Chuhao Chen, Xiaoyang Li, Yongqiang Zhang, Bo Wang, Yun Luo

**Affiliations:** 1 Third Affiliated Hospital of Sun Yat-sen University, Department of Urology, Guangzhou, 510630, People's Republic of China.; 2 Sun Yat-Sen Memorial Hospital, Sun Yat-Sen University, Department of Urology, Guangzhou, 510120, People's Republic of China.; 3 The First People's Hospital of Zhaoqing, Zhaoqing, 526000, People's Republic of China.

**Keywords:** CD204, Tumor-associated Macrophages, Clear cell renal cell carcinoma, Survival prognosis

## Abstract

**Purpose:** Tumor-associated macrophages (TAMs) play a crucial role in solid tumors and display varying characteristics depending on the specific tumor microenvironment (TME). The study investigated the presence and characteristics of TAMs in renal clear cell carcinoma (ccRCC) and assessed their influence on patient prognosis.

**Methods:** Immunohistochemistry (IHC) was used to identify CD204^+^ TAMs in a cohort of 72 patients with ccRCC. Kaplan-Meier survival analysis and log-rank test were used to evaluate the prognostic significance of CD204^+^ TAMs in each group. The TCGA-KIRC cohort was used to analyze the relationship between CD204 and immunity. The functions of CD204^+^ TAMs in the TCGA-KIRC cohort were analyzed through GO enrichment analysis. Immunofluorescence (IF) was conducted to confirm the positive effects of CD204 on regulatory T (Treg) cells and exhausted T (Tex) cells.

**Results:** There was a negative relation between high infiltration of CD204^+^ TAMs and both overall survival (OS) and progression-free survival (PFS) in ccRCC. A positive correlation was found between high-infiltrating CD204^+^ TAMs and distant organ metastasis, as well as lymph node metastasis. In the TCGA-KIRC cohort, the group with high expression of CD204 exhibited significant up-regulation of 120 genes as well as enrichment in the negative regulation of immunity. CD204 high-expression group showed up-regulation of Treg cells and Tex cells.

**Conclusion:** The presence of CD204^+^ TAMs in ccRCC is associated with a negative prognosis in patients. The high infiltration of CD204 promotes distant organ metastasis by aggerating Treg cells and Tex cells.

## 1. Introduction

Renal cell carcinoma (RCC) accounted for 90% of kidney cancer cases in 2020^1^. The primary cell types of RCC are clear cell renal cell carcinoma (ccRCC; 70%), papillary renal cell carcinoma (PRCC; 10-15%), and chromophobe RCC (5%)^2^. ccRCC is the most common form of kidney cancer. Previous research has shown a connection between ccRCC and mutations in the VHL gene, which is located on chromosome 3^3^. These mutations can cause resistance to chemotherapy and conventional radiotherapy, but they do respond to angiogenesis inhibitors. The immune infiltration score and T cell infiltration score of ccRCC are the highest among other common tumors, indicating that immunotherapy could potentially enhance the prognosis of ccRCC patients^3^. Therefore, immunotherapy, such as immune checkpoint therapy (ICT) and adoptive T cell therapy, is clinically combined with vascular endothelial growth factor tyrosine kinase inhibitors (TKIs) for the treatment of ccRCC, resulting in a significant effect. Before the introduction of ICT, increased localization of CD8^+^ T cells at the tumor margin within the tissue was associated with a decreased patient sensitivity to the immune response^4^, and Regulatory T cells (Tregs) may impair immune responses, resulting in suboptimal clinical outcomes of ICT^5^. High dose IL-2 therapy (HD IL-2) is an FDA-approved cytokine-based treatment with a low objective response rate (ORR) of only 12-20% in metastatic renal cell carcinoma. This suggests that there may be alternative pathways of immunosuppression that disrupt immune monitoring. The challenge is to identify new predictive factors and to develop prognostic models for advanced Renal Cell Carcinoma (RCC), aiming to enhance treatment strategies and enable personalized cancer therapy.

Factors influencing prognosis in ccRCC can be categorized into two main types: tumor-related factors, such as biomarkers associated with the tumor microenvironment, and host-related factors, including the clinical characteristics of the patient^6^. Developing drugs that target additional key prognostic biomarkers in ccRCC, beyond lymphocytes, could synergize with existing immunotherapies, potentially reducing immunosuppression and thus improving treatment effectiveness. Myeloid cells, including TAMs, myeloid-derived suppressor cells (MDSCs), tumor-associated neutrophils (TANs), and dendritic cells (DCs), are the predominant immune components in the TME. Dendritic cells play dual roles, functioning as both immunosuppressors and immunostimulators. They can present antigens to T cells and simultaneously provide immunomodulatory signals, either promoting immunity or inducing tolerance through cell-to-cell interactions and cytokines^7^, ^8^. DCs are classified into three main types: conventional DC1 (cDC1), conventional DC2 (cDC2), and plasma cell-like dendritic cells (pDC)^9^.cDC1 stimulates CD8^+^ cytotoxic T-cells^10^. cDC2 exhibits a heightened capability to elicit CD4^+^ T-cell responses^11^. During viral infections, pDC amplifies CD8^+^ T-cell responses by activating cDC1 via type 1 interferon (IFN1)^12^. MDSCs, consisting of myeloid progenitors, immature macrophages, immature granulocytes, and immature dendritic cells, are associated with unfavorable prognoses in various types of tumors^13^. In the initial stages of neoplasia, neutrophils exhibit both tumorigenic and antitumorigenic properties. Generally, high Tumor-Associated Neutrophil (TAN) levels correlate with a poorer response to chemotherapy and radiotherapy, although this trend does not hold for ovarian and gastric cancers^14^. In patients with locally advanced ccRCC, a higher baseline neutrophil-to-lymphocyte ratio (NLR) is indicative of a poorer prognosis^15^. In castration-resistant prostate cancer (CRPC) patients treated with enzalutamide, having an NLR >3 during treatment was associated with a poorer prognosis^16^. TAMs can differentiate into an inflammatory (M1) phenotype and an anti-inflammatory (M2) phenotype that promotes tumor immune evasion. M1-TAMS can produce pro-inflammatory cytokines, activate cytotoxic T-lymphocytes, and coordinate anti-tumor immune function 17. In contrast, selectively activated M2-TAMs can promote tumor progression by maintaining tumor cell viability and promoting angiogenesis and invasion through the expression of soluble mediators (e.g., ARG1 derivatives and TGFβ) as well as surface receptors (e.g., programmed death ligand 1 (PD-L1)) 18. Tumors that have a high presence of macrophages, especially those with pro-angiogenic characteristics, are associated with a poor prognosis and low overall survival 19. TAMs play a negative role in ICT and can prevent cytotoxic T cells from reaching tumor cells to exert killing 20. Cluster of differentiation 204 (CD204), also known as scavenger receptor-A (SR-A) or MSR1, is associated with poor prognosis in a variety of cancers 21-23. However, the role played by CD204^+^ TAMs in ccRCC is unknown.

## 2. Materials and Methods

### 2.1 Study population

A total of 72 ccRCC patients from Sun Yat-Sen Memorial Hospital, Sun Yat-Sen University, and 532 ccRCC patients from the TCGA-KIRC cohort were included in this study (*Table 1*). The follow-up time was measured from the date of their operation until the date of their death or the last follow-up. The statistical time interval ranged from 117 to 1912 days and 0 to 5907 days, with an average of 1111.8 days and 1373.8 days. The mortality rate during the follow-up period was 19.4% and 32.9%. The ages of the participants in the study ranged from 29 to 81 years and 26 to 90 years, with a mean age of 44.2 years and 58.8 years. Among the patients, there were 388 males and 216 females, accounting for 64.24% and 35.76% respectively. Additionally, there were 64 patients with tumors larger than 3 cm in diameter and 8 patients with tumors smaller than or equal to 3 cm in 72 patients, but in the TCGA-KIRC cohort, there was no relevant data. This represents proportions of 88.9% and 11.1%, respectively. Out of the total number of patients, 101 had distant organ metastases, while 501 did not. This corresponds to proportions of 16.72% and 82.95%, respectively. Tumor T-stages were categorized as T1-T2 and T3-T4, with 376 and 228 patients, respectively. The distribution of patients in each category was 62.25% and 37.75%. Out of the total number of patients, 579 had lymph node stage N0, while 25 had lymph node stage N1-N2, accounting for 95.86% and 4.14% respectively. The study selected overall survival (OS) as the outcome indicator, which is defined as the time from the date of surgery to the date of death or last follow-up.

### 2.2 Immunohistochemistry (IHC)

Formalin-fixed and paraffin-embedded samples were cut into 4μm sections, deparaffinized and hydrated, and then incubated with 0.3% H2O2 to block endogenous peroxidase activity. The antigens were heated using a microwave with a tris-EDTA buffer solution at pH 9.0. Sections were blocked using horse serum. After incubating overnight with a 1:2000 dilution of anti-human CD204 antibody (R&D Systems, Minneapolis, MN, USA) at 4°C, the sections were then treated with an anti-mouse antibody for 30 minutes. The secondary antibody was labeled using a DAB staining solution (Maixin Biologicals, Fuzhou, FOO, CHN), and sections were counterstained with hematoxylin (Sevier Biologicals Ltd, Wuhan, WH, CHN) and placed in PBS (Sevier Biologicals Ltd., Wuhan, WH, CHN).

### 2.3 Immunofluorescence (IF)

The paraffin tissues underwent the same process as immunohistochemistry, including deparaffinization, hydration, antigen retrieval, incubation with primary and secondary antibodies, and blocking with horse serum before each use of the primary antibody. After the secondary antibody incubation and washing steps, we used a multiplex fluorescent immunohistochemical five-color kit (Panovue Biological Technology, Beijing, BJ, CHN) for staining. The staining protocol included single staining for CD204, CD8α (1:3000 dilution, Thermo Fisher Scientific, Massachusetts, MA, USA)- PD1 (1:1000 dilution, Cell Signaling Technology, Boston, BSN, USA)-LAG3 (1:3000 dilution, Cell Signaling Technology, Boston, BSN, USA), and CD8-CTLA4 (1:1000 dilution, Cell Signaling Technology, Boston, BSN, USA)-CD28 (1:10000 dilution, R&D Systems, Minneapolis, MN, USA). After incubating the slides with primary and secondary antibodies, a mounting medium containing DAPI (Beijing Solarbio Science & Technology Co., Ltd, Beijing, BJ, CHN) is used to load the slides.

### 2.4 Digital quantification of stroma

The sections were scanned using a pathology slide scanner in panoramic view. Then, 3-5 independent high magnification (×400) fields of view, each with an area size of 0.0768 mm^2^, were selected for cell counting using Case Viewer 2.4 (3DHISTECH, Budapest, BP, HU), respectively. Finally, the number of CD204^+^ TAMs was counted and averaged to determine the expression density of CD204^+^ TAMs in the tumor tissue (mean number of positive cells/area).

### 2.5 Statistical analysis

The statistical analyses were conducted using SPSS software (Statistical Product and Service Solutions 22, IBM, Cary, NC, USA). All p-values were two-sided, indicating significant differences (*P* < 0.05). Chi-square tests were conducted for categorical data. Survival analysis was conducted using the Kaplan-Meier method and log-rank test. The comparison between groups with high and low CD204 expression in the SYSU data set was performed using the Welch t-test, accounting for normal distribution but unequal variances. For the TCGA-KIRC data set, group differences were analyzed using the Wilcoxon rank sum test.

### 2.6 Bioinformatics analysis

Based on CD204 expression profile data from the TCGA-KIRC cohort, CD204 expression was divided into high and low-expression groups using the median CD204 expression. Differential gene analysis was performed using the limma package. The screening criteria were set at a significance level of *P*<0.05 and |log2 (Fold change) |≥0.80.

## 3. Results

### 3.1 Prognostic value of CD204^+^TAMs infiltration for poor clinical outcomes in ccRCC patients

**Figure 1:**
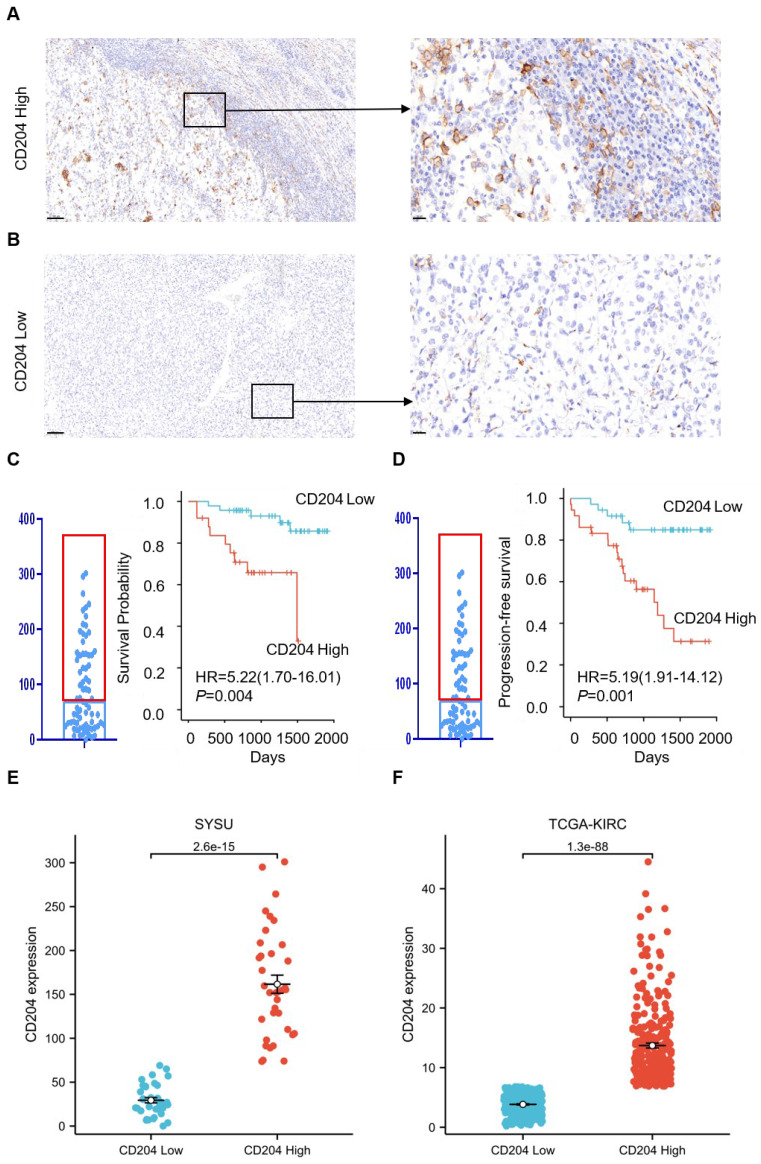
Predictive value of CD204^+^TAM infiltration abundance for ccRCC patients (A, B) Representative plots of CD204^+^ TAMs with high and low infiltration in ccRCC; scale bar: 100um (left), 20um (right). (C, D) Kaplan-Meier curves of overall survival (OS) and Progression-survival (PFS) based on CD204+ TAMs infiltration: log-rank analysis comparison of survival between the CD204 High group (n=36) and the CD204 Low group (n=36). (E, F) Comparison of CD204 density between high and low groups in SYSU and TCGA-KIRC cohorts, respectively.

CD204 is a surface marker for M2 TAMs, but its role in renal cancer has received limited research attention. To deeply investigate the potential role of CD204^+^ TAMs in clear cell renal cell carcinoma, we analyzed the expression of CD204 in 72 ccRCC tumor specimens with different clinicopathological features. The results showed varying levels of expression of CD204^+^ TAMs in all ccRCC samples. The cells were mainly located in the tumor stroma, with some infiltrating the cancer nest and accumulating along the invasive tumor margin (*Fig. 1A-B*). The results indicate that CD204^+^ TAMs are crucial in the TME of renal cancer and may have a significant impact on regulating the immune response to the tumor. To investigate the relationship between CD204^+^ TAMs infiltration and clinical outcome in ccRCC, we performed an analysis using Kaplan-Meier survival curves and log-rank methods. In this case, the median was used as the cutoff value to divide the 72 samples into a high infiltrate group (n=36) and a low infiltrate group (n=36). In renal cancer, we observed a negative correlation between the infiltration of high intratumoral CD204^+^ TAMs and OS (HR=5.22, *P*=0.004) and PFS (HR=5.19, *P*=0.001) (*Fig. 1C-D*). In the SYSU cohort, the IQR of CD204 density was 27 (18.58, 44.83) in the low-expression group and 153.83 (108.83, 198.88) in the high-expression group. In the TCGA-KIRC cohort, the IQR was 3.93 (2.51, 2.63) for the low-expression group and 11.86 (7.24, 8.80) for the high group. The difference in CD204 density between these groups in both SYSU and TCGA-KIRC cohorts was statistically significant (*P*<0.001) (*Fig. 1E-F*).

### 3.2 Relationship between CD204^+^ TAMs infiltration level and clinical characteristics of different ccRCC patients

**Figure 2:**
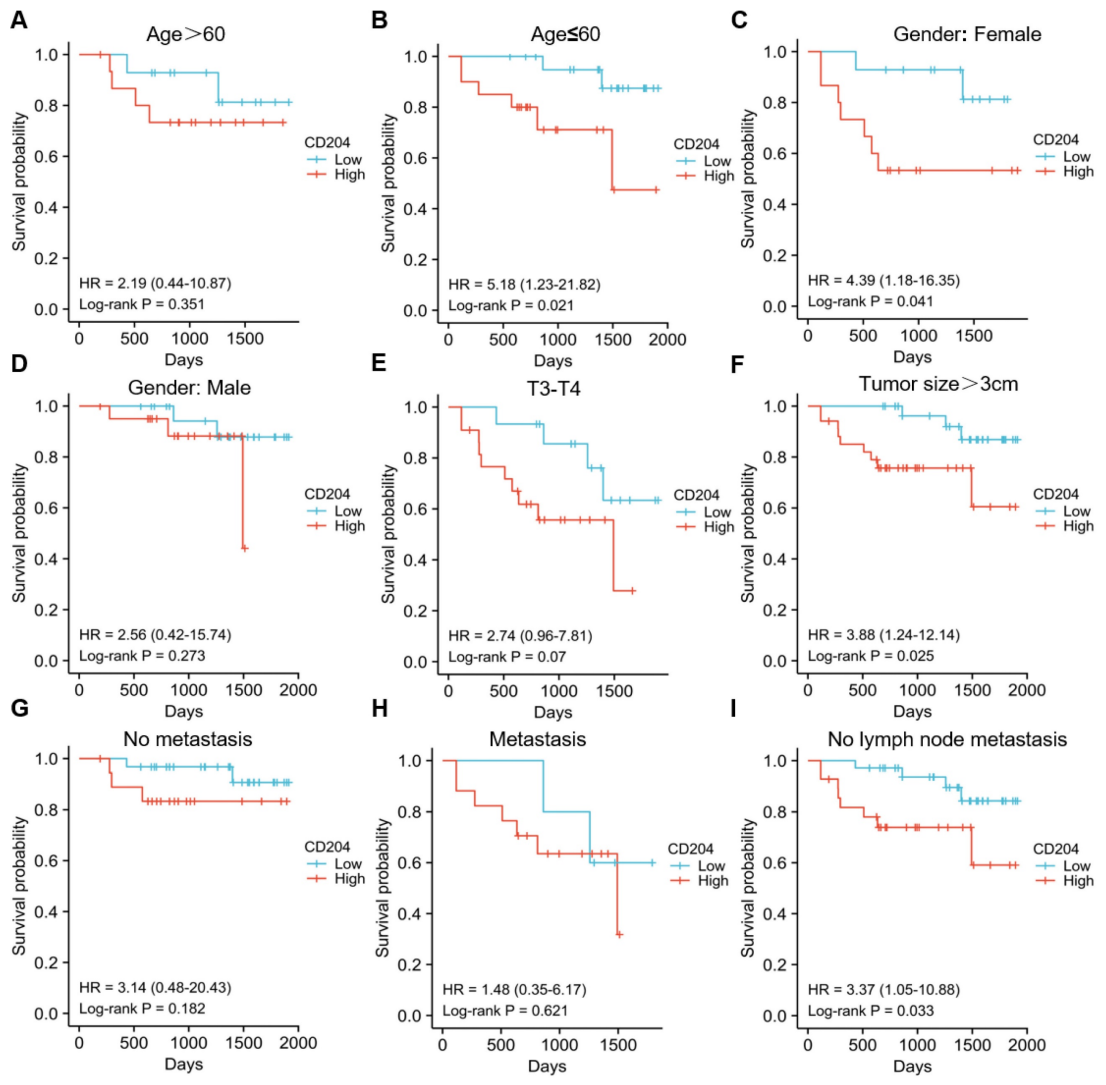
Accumulation of CD204^+^ TAMs in tissues of different subgroups of ccRCC patients predicts poor prognosis Kaplan-Meier survival curve and log-rank test were utilized to analyze the prognostic significance of infiltrating CD204^+^ TAMs in each group. Patients were stratified by age (A, B), gender (C, D), tumor stage (E), tumor size (F), distant organ metastasis (G, H), and lymph node metastasis (I). The red group represents CD204^+^ TAMs with high infiltration, while the blue group represents CD204^+^ TAMs with low infiltration.

**Figure 3:**
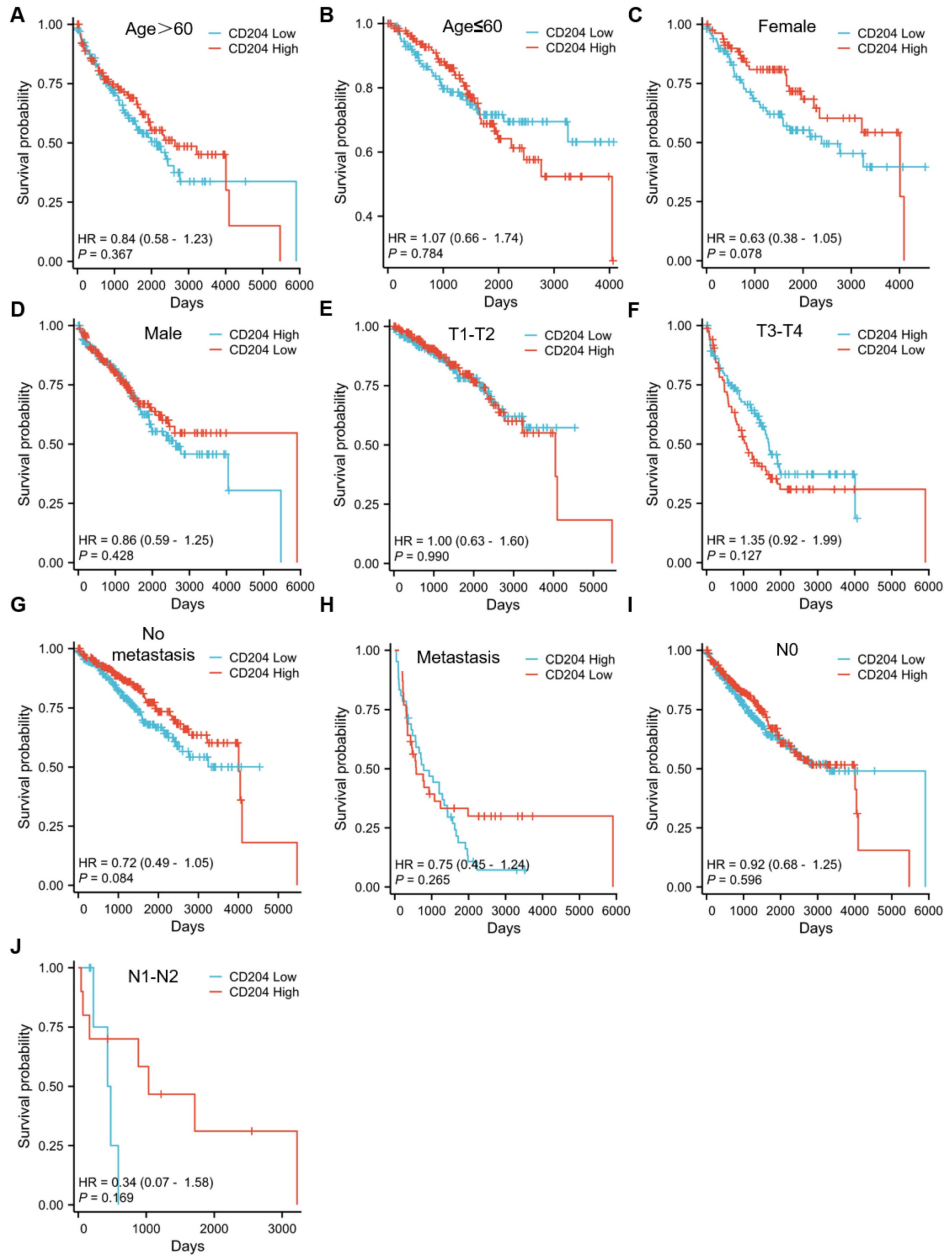
The accumulation of CD204^+^ TAMs in various subgroups of ccRCC patients is indicative of a poor prognosis. Kaplan-Meier survival curves and log-rank tests were employed to assess the prognostic relevance of CD204^+^ TAMs infiltration in each subgroup. Patients were categorized based on age (A, B), gender (C, D), tumor stage (E, F), presence of distant organ metastasis (G, H), and lymph node involvement (I, J).

To further assess the prognostic significance of CD204^+^ TAMs in various subgroups of ccRCC, patients were analyzed separately by age (*Fig. 2A-B*), gender (*Fig. 2C-D*), tumor T stage (*Fig. 2E*), tumor size (*Fig. 2F*), distant organ metastasis (*Fig. 2G-H*), and lymph node metastasis stage (*Fig. 2I*). No fatal events occurred in tumor stages T1-T2, and there were too few patients with tumor diameter ≤3cm and lymph node stage N1-N2 to be excluded. The infiltration level of CD204^+^ TAMs had prognostic value in specific subgroups, including age ≤60 years (*P*=0.021), female (*P*=0.041), tumor >3cm (*P*=0.025), and no distant lymph node metastasis (*P*=0.033). CD204^+^ TAMs may be a significant prognostic factor in ccRCC cases, regardless of the risk group. We analyzed the correlation between CD204 expression in the TCGA-KIRC cohort and variables such as age, gender, and TNM stage, but found no statistically significant associations (*Fig. 3*). Finally, we conducted a Chi-square test analysis to assess the correlation between CD204^+^ TAM density and clinicopathologic variables (*Table 2*). The study found a positive correlation between the density of CD204^+^ TAMs and distant organ metastasis (χ^2^=9.425, *P*=0.002), as well as lymph node metastasis (χ^2^=6.222, *P*=0.013).

### 3.3 Analysis of differentially expressed genes (DEGs) in CD204 and their correlation with immune cells

**Figure 4:**
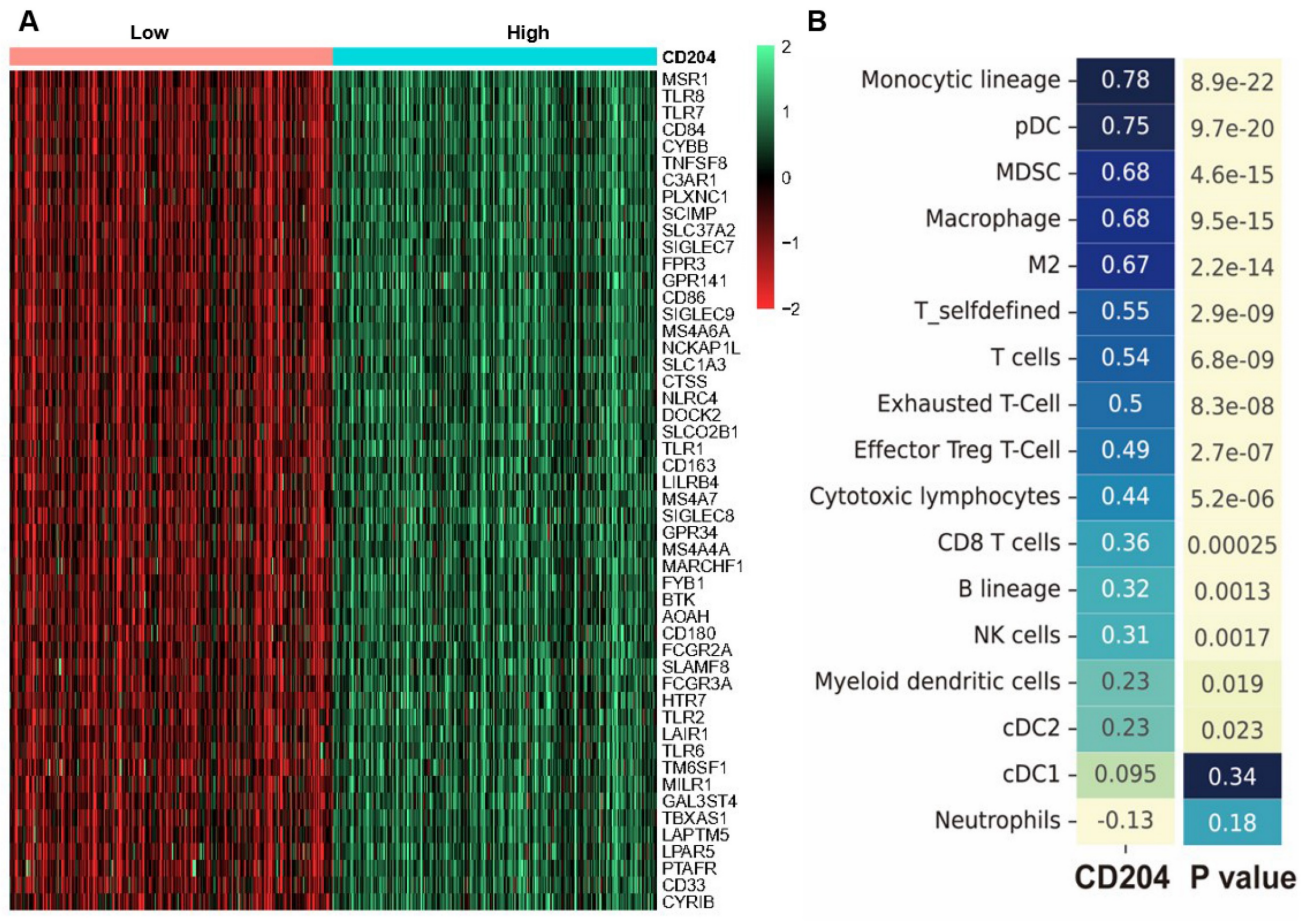
Analysis of CD204 differentially expressed genes and correlation of CD204 with immune cells (A) Limma package analysis of sequencing data from 532 patients in the TCGA-KIRC cohort. The screening criteria for CD204 differentially expressed genes were a significance level of *P*<0.05 and |log2 (Fold change)|≥0.8. The CD204 high-expression group (blue) in the TCGA-KIRC cohort showed up-regulation of 120 genes and no down-regulated genes compared to the CD204 low-expression group (red). The figure displays the top 50 differentially expressed genes. (B) Immune cells related to CD204.

We examined the genes that were expressed differently between the two groups with high and low CD204 expression in ccRCC. The results revealed that the CD204 high expression group in the TCGA-KIRC cohort had 120 up-regulated genes and no down-regulated genes compared to the CD204 low expression group. The top 50 significantly up-regulated differential genes (*Fig. 4A*) were selected, including LAIR1, TLR1/2/6/7/8, CD84, CD33, DOCK2, CYBB, and other genes.

To analyze the association between CD204^+^ TAMs and immune cells, we used the MCP counter algorithm to determine the relative abundance of 17 immune cells in the TCGA-KIRC cohort. We then correlated these immune cells with CD204. In the TCGA-KIRC cohort, strong correlations with CD204 were observed in monocytes (Spearman, r=0.78, *P*<0.001) and plasma cell-like DCs (Spearman, r=0.75, *P*<0.001). Moderate correlations were found in macrophages, MDSCs (Spearman, r=0.68, *P*<0.001), T cells (Spearman, r=0.54, *P*<0.001), Tex cells (Spearman, r=0.5, *P*<0.001), and Treg cells (Spearman, r=0.49, *P*<0.001). Weak correlations were observed in CD8^+^ T cells, B lymphocytes, and cDC2. CD204 was unrelated to cDC1 and neutrophils (*Fig. 4B*).

### 3.4 Functional mechanisms of CD204^+^ TAMs and poor prognosis in ccRCC

**Figure 5:**
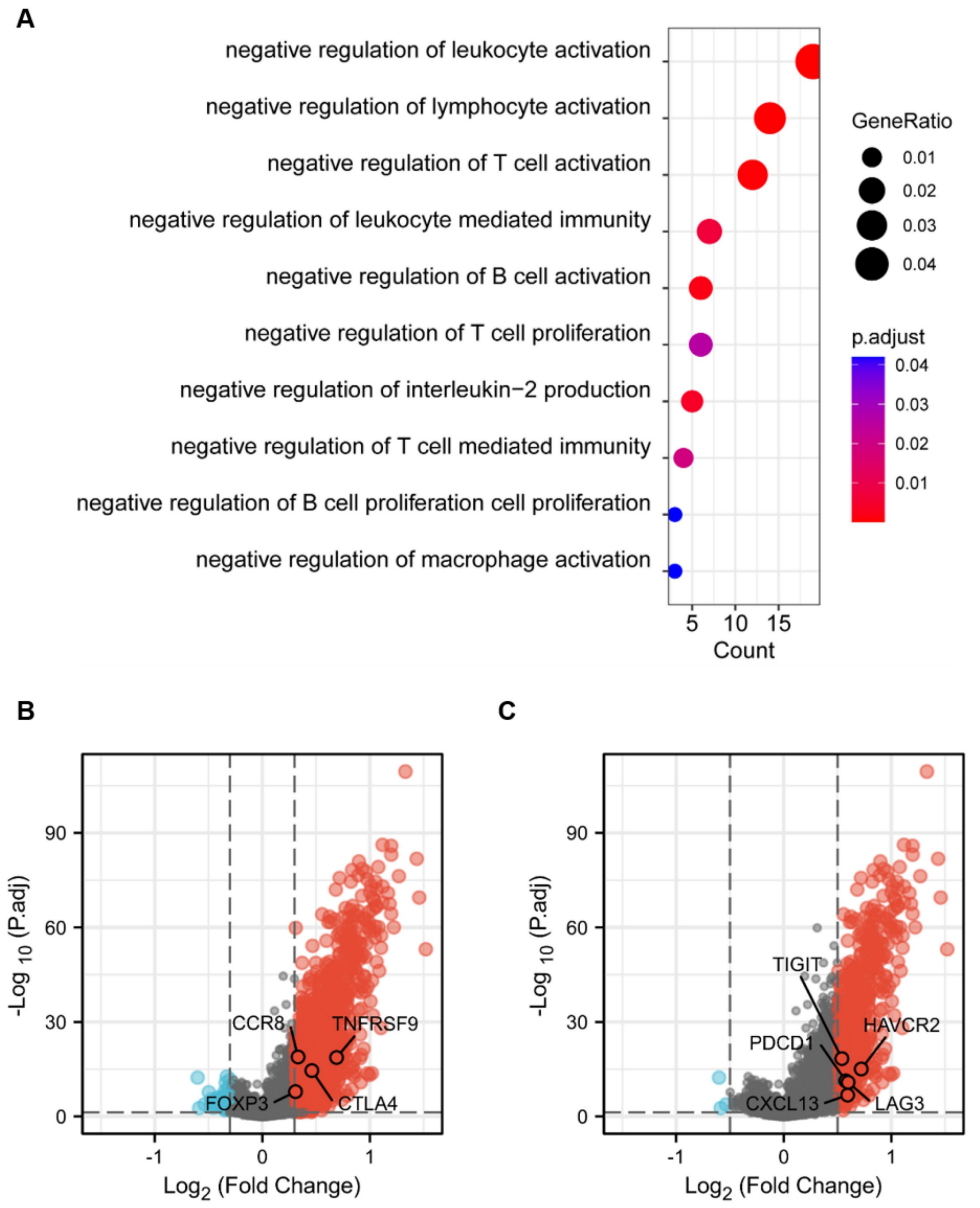
Possible functions of CD204^+^ TAMs in ccRCC (A) GO pathway enrichment analysis of differentially expressed genes in the TCGA-KIRC cohort revealed that the high-expression group, as opposed to the CD204 low-expression group, was enriched for negative regulation of leukocyte, T cell, and B cell activation, negative immunological regulation involving lymphocytes. (B) Volcano plot: Markers representing the regulatory T cells, including CCR8, TNFRSF9, CTLA4, and FOXP3, were upregulated in the CD204 high-expression group. CCR8, TNFRSF9, CTLA4, and FOXP3 markers representing exhausted T cells, are increased in the CD204 high-expression group. (C) Volcano plot: TIGIT, PDCD1, HAVCR2, LAG3, and CXCL13 markers, which represent exhausted T cells, are upregulated in the CD204 high-expression group.

To explore the functional mechanism of CD204^+^ TAMs in ccRCC, we performed GO enrichment analysis on DEGs in the TCGA-KIRC cohort. The results showed that the group with high CD204 expression had a greater concentration of negative regulation of leukocyte/T cell/B cell activation and negative regulation of immunity involving lymphocytes, compared to the group with low CD204 expression (*Fig. 5A*). This suggests that the CD204 high-expression group exhibited greater immunosuppressive activity within ccRCC tissues, creating an immunosuppressive microenvironment that could potentially facilitate tumor progression. We analyzed genes associated with immunosuppression in Tex and Treg cells to further investigate downregulated gene changes. It was visually understood from volcano plots where some genes such as CCR8, TNFRSF9, CTLA4, and FOXP3, which are associated with Treg cells, and related indicators (TIGIT, PDCD1, HAVCR2, LAG3, CXCL13) that suggest exhaustion of T cell function, were up-regulated in the high-expressing CD204 group (*Fig. 5B-C*).

### 3.5 CD204^+^ TAMs and the recruitment of exhausted T cells and regulatory T cells

**Figure 6:**
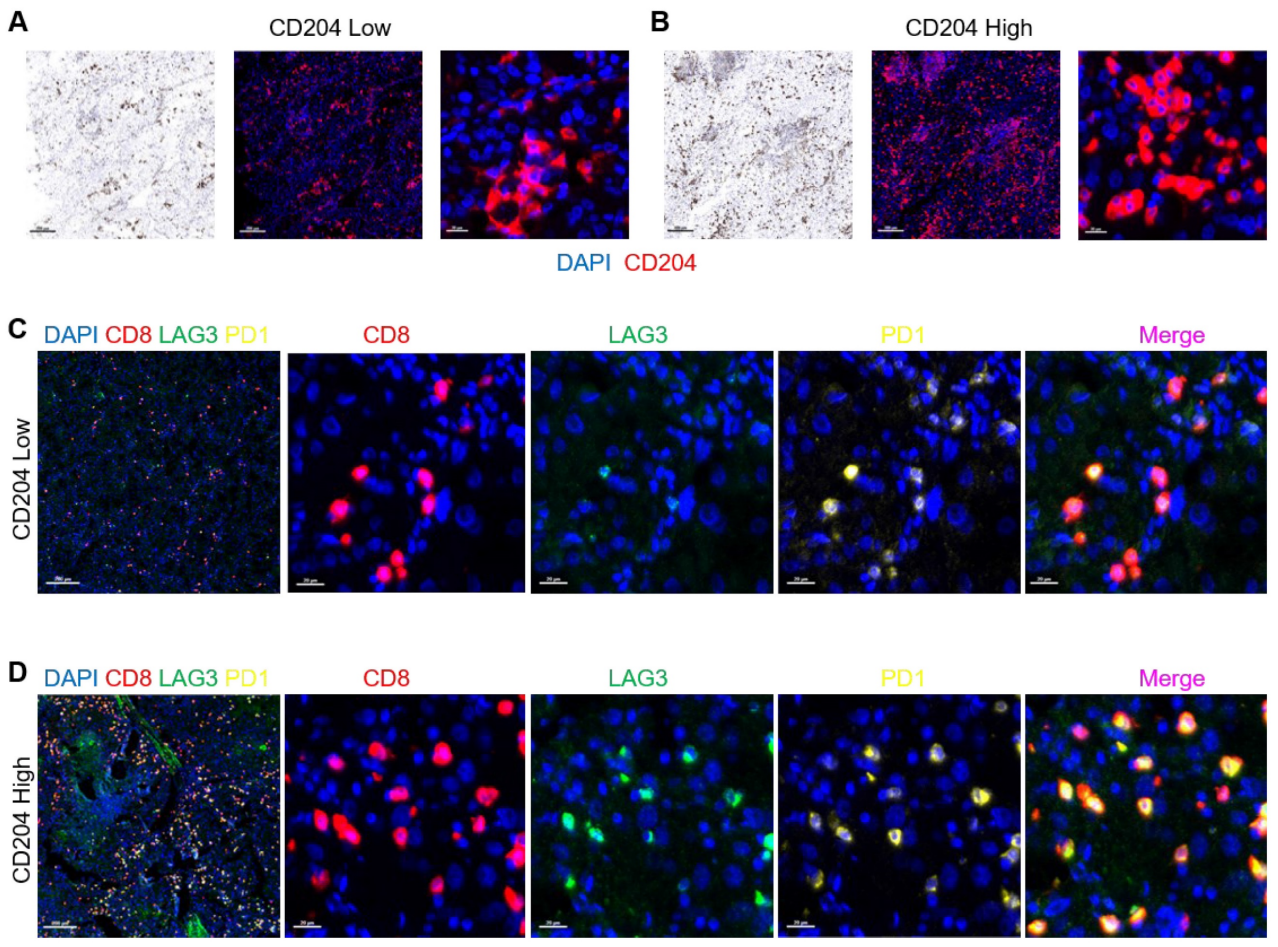
CD204^+^ TAMs and recruitment of exhausted T cells (A, B) Expression of CD204 in tissues from two selected cases (CD204 low-expression and high-expression respectively). DAPI (blue); CD204(red). (C, D) Tex cell infiltration represented by PD1^+^ LAG3^+^ CD8^+^ was more frequent in ccRCC tissues with high CD204 expression compared to those with low expression. Tex: exhausted T. DAPI (blue); CD8 (red); PD1 (yellow); LAG3 (green). Scale bar: 200um; 20um.

We conducted a study to examine the correlation between CD204 and Tex and Treg cells in ccRCC. After staining the tissues of two selected cases, we found that tissues with high CD204 expression had a higher infiltration of PD1^+^ LAG3^+^ CD8^+^ T cells compared to those with low expression (*Fig. 6*).

**Figure 7:**
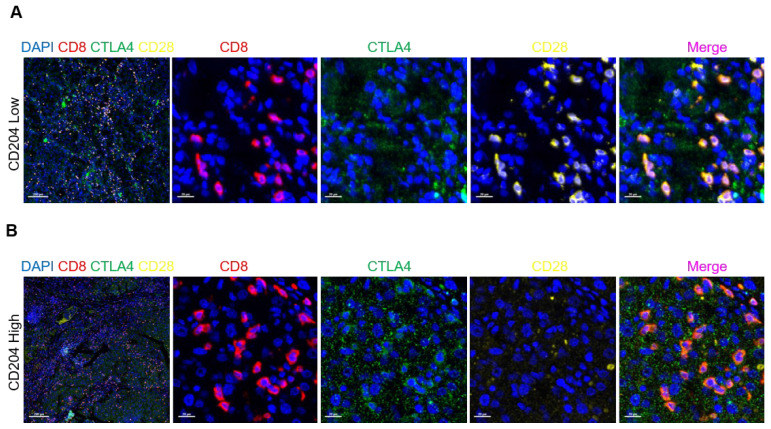
CD204^+^ TAMs recruited Treg cells in ccRCC (A, B). Tissues with low CD204^+^ TAMs infiltration exhibited decreased infiltration of CTLA4^+^CD8^+^ Treg cells and increased infiltration of CD28^+^CD8^+^ effector T cells, compared to tissues with high CD204^+^ TAMs infiltration. Treg refers to regulatory T cells. Immunofluorescence staining: DAPI (blue), CD8 (red), CTLA4 (green), CD28 (yellow). Scale bars represent 200 µm and 20 µm.

We also observed that CTLA4, which represents Treg cells, had a higher infiltration compared to CD28, which stimulates the proliferative activation of T cells in the high-expression group (*Fig. 7*).

## 4. Discussion

CD204/MSR1 is a critical marker of TAMs and has been associated with cancer progression and poor prognosis. A higher ratio of CD8^+^ T cells to CD204^+^ TAMs is associated with a favorable postoperative prognosis in prostate cancer 24. In thymic carcinoma, the ratio of CD8^+^ T cells/CD204^+^ TAMs and CD20^+^ B cells/CD204^+^ TAMs correlate with prognostic outcomes in the stromal region 25. In non-small cell lung cancer (NSCLC) 26 and muscle-invasive bladder cancer 27, a higher number of CD204^+^ TAMs in the stromal area of the tumor is associated with various clinicopathological factors, poor prognosis, and shorter survival time. The presence of a high number of CD204^+^ TAMs has been linked to the aggressive nature of upper urinary tract cancers 28. The analysis of immune cells infiltrating osteosarcoma metastases indicates that MSR1 expression is associated with resistance to metastasis and improved overall survival rates 29. In this study, we confirmed that a high density of CD204^+^ TAMs in renal clear cell carcinoma is associated with shorter overall survival time and progression-free survival time. Additionally, we observed that high infiltration of CD204^+^ TAMs is correlated with age, gender, tumor size, and lymph node metastasis, which are pathological features. Our findings suggest that tumor-infiltrating MSR1/CD204 is a biomarker that is associated with the prognosis of ccRCC.

There is increasing evidence that macrophages generate a unique environment that supports tumor progression 30. TAMs can inhibit cytotoxic T-cell activity and promote the growth of regulatory T-cells, leading to immune evasion and tumor proliferation 31, 32. Compared to brain metastases and primary lung tumors, there is a notable increase in the number of MSR1^+^ TAMs within the TME of metastatic tumors, while other cellular components of the immune system do not show a similar increase 33. The quantity of MSR1^+^ macrophages is significantly influenced by lymphangiogenesis in pancreatic tissues with lymph node metastases 34. In addition, CD204^+^ TAMs present greater levels of IL-10 and MCP-1, which are involved in the accumulation, migration, and polarization of M2 macrophages 35. A study demonstrated that the stimulation of macrophages using a conditioned medium from breast cancer cell lines leads to an upregulation of CD204 expression 23, This approach only confirmed the impact of the tumor on macrophages but did not consider the role of CD204^+^ TAMs within the TME. We analyzed the TCGA database to investigate the influence of CD204^+^ TAMs on the ccRCC tumor microenvironment. Our findings revealed that high CD204 expression was correlated with elevated levels of regulatory T cell-related genes, as well as exhausted T cell-related genes. CD204^+^ TAMs contribute to the development of an immunosuppressive microenvironment in ccRCC by depleting cytotoxic T cells and facilitating the accumulation of Treg cells, thereby promoting tumor growth and progression. The immune microenvironment analysis of ccRCC tissues expressing CD204 revealed that Tex cells and Treg cells had higher infiltration rates in tissues with high-density CD204 expression compared to those with low-density CD204 expression.

This study utilized immunohistochemical analysis to investigate the role of CD204^+^ TAMs as potential predictive markers for ccRCC, providing limited insights into the functions and mechanisms of CD204/MSR1 within TME. While the laboratory methods did not represent a significant breakthrough, the incorporation of bioinformatics suggested that the infiltration of CD204^+^ TAMs in ccRCC negatively impacts the TME. To substantiate these findings, further studies involving cellular experiments, animal models, or organoid cultures that mimic the TME with overexpressed CD204 in macrophage cell lines would be necessary.

In summary, CD204/MSR1 can serve as a biomarker for predicting poor survival in ccRCC. The specific mechanism by which CD204^+^ TAM promotes tumor progression through its impact on other immune cells remains unclear.

## Funding

This work was supported by the National Natural Science Foundation of China [grant number 82072831]; the Science and Technology Planning Project of Guangzhou [grant number 202102010235]; the City and Hospital Joint Funding Project [grant number KTP2020335]; and the Foundation of the 3rd Affiliated Hospital of Sun Yat-sen University [No grant number].

## Author statements

All authors approved the final manuscript and the submission to this journal.

Y.X.X. wrote the main manuscript text, finished the experiments and statistics, and created the figures and tables, G.J.T. analyzed the TCGA data when we needed modifications, B.W. provided the experimental ideas, P.X. prepared Figure 3 and the bioinformatics analysis support, X.Z. completed the insertion of references, C.H.C. polished the manuscript, X.Y.L. and Y.Q.Z. were involved in some of the experimental steps, and Y.L. provided grant support. All authors reviewed the manuscript.

## Institutional review board statement

The study was conducted in accordance with the Declaration of Helsinki, and approved by the Ethics Committee of the Third Affiliated Hospital of Sun Yat-sen University and Sun Yat-Sen Memorial Hospital of Sun Yat-Sen University.

## Figures and Tables

**Table 1:** Baseline patient data table. The study analyzed data from 72 patients with ccRCC at Sun Yat-Sen Memorial Hospital, Sun Yat-Sen University, and 532 patients from TCGA-KIRC, including information on age, gender, tumor size, distant organ metastasis, tumor stage, lymph node metastasis, and follow-up time.

Variables	Percentage %	
	SYSU	TCGA-KIRC
Number	72	532
Age (average, range)	44.2 (29-81)	58.8 (26-90)
Gender (male/female)	43/29 (59.7/40.3)	345/187 (64.8/35.2)
Tumor size (≤3cm/>3cm)	8/64 (11.1/88.9)	unknown
Metastasis (no/yes/unknown)	50/22/0 (69.4/30.6/0)	451/79/2 (84.8/14.8/0.4)
Tumor stage (T1-T2/T3-T4)	35/37 (47.2/52.8)	341/191 (64.1/35.9)
Lymph node metastasis (N0/N1-N2)	63/9 (87.5/12.5)	516/16 (97.0/3.0)
Follow-up time/day (average, range)	1111.8 (117-1912)	1373.8 (0-5907)

**Table 2:** The correlation between CD204^+^ TAM density and clinicopathologic variables. According to the Chi-square test, there was a positive correlation between CD204^+^ TAM density and distant organ metastasis (χ^2^=9.425, *P*=0.002) and lymph node metastasis (χ^2^=6.222, *P*=0.013). However, CD204^+^ TAM density was not found to be related to age, gender, tumor size, or tumor stage.

Variables	CD204^+^ Low (n=36)	CD204^+^ High (n=36)	χ^2^	*P*-value
Age (≤60/>60)	22 (52.38%) 14 (46.67%)	20 (47.62%) 16 (53.33%)	0.229	0.633
Gender (female/male)	14 (48.28%) 22 (51.16%)	15 (51.72%) 21 (48.84%)	0.058	0.810
Tumor size (>3cm/≤3cm)	30 (46.88%) 6 (75.00%)	34 (53.13%) 2 (25.00%)	2.250	0.134
Metastasis (yes/no)	5 (22.73%) 31 (62.00%)	17 (77.27%) 19 (38.00%)	9.425	0.002
Tumor stage (T3-T4/T1-T2)	14 (40.00%) 22 (59.46%)	21 (60.00%) 15 (40.54%)	2.724	0.099
(N1-N2/N0)	1 (11.11%) 35 (55.56%)	8 (88.89%) 28 (44.44%)	6.222	0.013
